# Impact of the National Reimbursement Drug List Negotiation Policy on Accessibility of Anticancer Drugs in China: An Interrupted Time Series Study

**DOI:** 10.3389/fpubh.2022.921093

**Published:** 2022-07-01

**Authors:** Hong Zhu, Jingmin Zhu, Yingyu Zhou, Linghan Shan, Cui Li, Yu Cui, Zheng Kang, Mingli Jiao, Huan Liu, Lijun Gao, Qunhong Wu, Yanhua Hao

**Affiliations:** ^1^Department of Health Policy, Health Management College, Harbin Medical University, Harbin, China; ^2^Department of Social Medicine, School of Public Health, Harbin Medical University, Harbin, China; ^3^Department of Pharmacy Administration, Humanities and Social Science College, Harbin Medical University, Harbin, China; ^4^Department of Epidemiology and Public Health, University College London, London, United Kingdom; ^5^Science and Technology Development Center, Chinese Pharmaceutical Association, Beijing, China; ^6^Department of Medical Procurement, The Fourth Affiliated Hospital, Harbin Medical University, Harbin, China

**Keywords:** accessibility, availability, cost, anticancer drugs, National Reimbursement Drug List Negotiation, China

## Abstract

**Objective:**

Since 2016, the Chinese government has been regularly implementing the National Reimbursement Drug List Negotiation (NRDLN) to improve the accessibility of drugs. In the second round of NRDLN in July 2017, 18 anticancer drugs were included. This study analyzed the impact of the NRDLN on the accessibility of these 18 anticancer drugs in China.

**Methods:**

National hospital procurement data were collected from 2015 to 2019. As measurements of drug accessibility, monthly average of drug availability or defined daily dose cost (DDDc) was calculated. Interrupted time series (ITS) analysis was employed to evaluate the impact of NRDLN on drug accessibility. Multilevel growth curve models were estimated for different drug categories, regions or levels of hospitals.

**Results:**

The overall availability of 18 anticancer drugs increased from about 10.5% in 2015 to slightly over 30% in 2019. The average DDDc dropped from 527.93 CNY in 2015 to 401.87 CNY in 2019, with a reduction of 23.88%. The implementation of NRDLN was associated with higher availability and lower costs for all 18 anticancer drugs. We found an increasing level in monthly drug availability (β_2_ = 2.1126), which ascended more sharply after the implementation of NRDLN (β_3_ = 0.3656). There was a decreasing level in DDDc before July 2017 (β_2_ = −108.7213), together with a significant decline in the slope associated with the implementation of NRDLN (β_3_ = −4.8332). Compared to Traditional Chinese Medicines, the availability of Western Medicines was higher and increased at a higher rate (β_3_ = 0.4165 vs. 0.1108). Drug availability experienced a larger instant and slope increase in western China compared to other regions, and in secondary hospitals than tertiary hospitals. Nevertheless, regional and hospital-level difference in the effect of NRDLN on DDDc were less evident.

**Conclusion:**

The implementation of NRDLN improves the availability and reduces the cost of some anticancer drugs in China. It contributes to promoting accessibility of anticancer drugs, as well as relieving regional or hospital-level disparities. However, there are still challenges to benefit more patients sufficiently and equally. It requires more policy efforts and collaborative policy combination.

## Introduction

Cancer is one of the leading causes of death and disability worldwide (1, 2). In China, as reported by National Central Cancer Registry, there were ~4.06 million new cancer cases and 2.41 million cancer deaths in 2016 (3). Meanwhile, anticancer drug costs have imposed heavy burdens on patients and their families, as well as China's economy and healthcare system. In 2018, global expenditure on anticancer drugs was as high as $6.3 billion USD, accounting for around 70% of total expenditure on cancer treatment (4).

In order to promote equitable access to cancer care and ensure its affordability, the international society and governments are taking actions to ensure adequate supplies and access to safe, affordable, and effective quality anticancer drugs (1, 5). For example, the American Society of Clinical Oncology (ASCO) assessed a variety of cost-cutting proposals, such as allowing Medicare to negotiate the prices of anticancer drugs, legalizing anticancer drug importation, and bundled payment programs (6).

However, according to the 2018 Access to Medicine Index, anticancer drug accessibility was low in developing countries (7). Major challenges remain in increasing population's access to anticancer drugs, especially in resource-poor settings and low- and middle-income countries, mainly due to lack of government reimbursements, insufficient budget allocations for healthcare, the unavailability of quality-assured generic and biosimilar medicines, shortages, and so on (1, 7, 8).

In China, the accessibility of innovative anticancer drugs is dim. Only 6 of 49 innovative anticancer drugs launched across the world between 2010 and 2014 are available in China. In terms of global accessibility to innovative anticancer drugs, China ranks far lower than developed countries, and is also behind India (9). In this regard, Chinese government has committed a series of policies and practices in recent years to increase the accessibility of anticancer drugs, such as tariff exemptions on imported anticancer drugs, centralizing the government procurement of anticancer drugs, and the long-term strategy of controlling anticancer drug pricing (10).

Incorporating more anticancer drugs into the National Reimbursement Drug List Negotiation (NRDLN) is one of China's recent significant efforts to reduce drug price, and improve its accessibility and affordability. The NRDLN policy was initiated through a collaboration between the National Healthcare Security Administration (NHSA) and pharmaceutical companies in 2016. Whether one drug enters national procurement list is determined through a centralized strategic price negotiation when candidate drugs are assessed comprehensively in terms of safety, efficacy, clinical need, reference price and comparative value (11). After the negotiation, the drug procurement price is largely reduced and based on further economic assessment, some of them enter the national reimbursement drug list (12). Along with the procurement price, payment standards, reimbursement level of listed drugs and detailed reimbursement restrictions (such as indications, treatment duration, number of doses, etc.) are determined by NRDLN as well (13). Following the NRDLN, public hospitals must purchase listed drugs *via* provincial procurement websites based on the negotiated prices, and then the reimbursement is paid by national medical insurance fund (14). In July 2017, 36 drugs were added to the list, 18 of which were anticancer drugs for major cancers, such as lung cancer, rectum cancer and leukemia (15).

In some recent studies, as measured by availability and cost, changes in the accessibility of anticancer drugs are investigated (16–24). In regard to the NRDLN, evidence has been provided on the changes in cost, availability, affordability, and clinical uses of listed drugs in specific regions and provinces of China (25–29). However, there is still a lack of longer-term nationwide empirical evidence on the impact of the implementation of NRDLN on the accessibility of anticancer drugs, nor is there a comprehensive or stratified picture of the current national situation in China.

In this study, we used national hospital procurement data over a 5-year period to explore the accessibility of 18 anticancer drugs, which were added to the national reimbursement drug list in 2017. Therein, we conducted an interrupted time series (ITS) analysis and employed a multilevel growth curve model to quantify changes in availability and cost associated with the NRDLN. It was hypothesized that the NRDLN would increase the availability and reduce costs of anticancer drugs.

## Materials and Methods

### Data Source

We collected data from the Chinese Medicine Economic Information Network (CMEI), which was established and still managed by the Science and Technology Development Center of Chinese Pharmaceutical Association. The CMEI is one of the largest medical-economic and drug information service platforms, covering over 1,500 public hospitals in China (30). It provides hospital information and monthly drug procurement data for each hospital, including the drug category, generic name, code, dose, brand-name producer, indications, procurement volume, and cost (procurement price). However, we only have access to the average of national drug availability and cost in each month, which provides us with time series data.

### Sample and Data Collection

We focused on 18 anticancer drugs incorporated into the national reimbursement drug list in July 2017, including 15 Western Medicines and 3 Traditional Chinese Medicines. Detailed information of these 18 anticancer drugs is listed in Table 1.

**Table 1:** Characteristics of 18 investigated anticancer drugs.

**No**.	**Generic name**	**Code**	**Dosage form and strength**	**Indications**	**Market approval date on NMPA^**#**^**
1	Abiraterone	L02BX03^▴^	250 mg (Tab)	Prostatic cancer	May 2015
2	Apatinib	L01XE07^▴^	250 mg, 375 mg, 425 mg (Tab)	Gastric adenocarcinoma	October 2014
3	Bevacizumab	L01XC07^▴^	100 mg/4 ml (Inj)	Colorectal cancer, and Non-small-cell lung cancer (NSCLC)	February 2010
4	Bortezomib	L01XX32^▴^	3.5 mg, 1 mg (Inj)	Multiple myeloma (MM), and lymphoma	February 2005
5	Chidamide	XL01XX^*^	5 mg (Tab)	Peripheral T cell lymphomas (PTCL)	December 2014
6	Erlotinib	L01XE03^▴^	150 mg, 100 mg (Tab)	NSCLC	April 2006
7	Everolimus	L04AA18^▴^	5 mg, 2.5 mg (Tab)	Renal cell carcinoma, endocrine neoplasia, and angiomyolipoma (AML)	January 2013
8	Fulvestrant	L02BA03^▴^	250 mg/5 ml (Inj)	Breast cancer	June 2010
9	Lapatinib	L01XE07^▴^	250 mg (Tab)	Breast cancer	January 2013
10	Lenalidomide	L04AX04^▴^	10 mg, 25 mg (Tab)	MM	January 2013
11	Nimotuzumab	XL01XC^*^	50 mg/10 ml (Inj)	Nasopharyngeal carcinoma(NPC)	April 2008
12	Recombinant human endostatin	XL01XX^*^	15 mg/2.4 ×10^5^ U/3 ml (Inj)	NSCLC	September 2005
13	Rituximab	L01XC02^▴^	100 mg/10 ml, 500 mg/50 ml (Inj)	Non-Hodgkin's lymphoma (NHL)	April 2000
14	Sorafenib	L01XE05^▴^	200 mg (Tab)	Renal cell carcinoma, hepatocellular carcinoma(HCC),and thyroid carcinoma	September 2006
15	Trastuzumab	L01XC03^▴^	440 mg/20 ml (Inj)	Breast cancer, and gastric cancer	September 2002
16	Compound realgar natural indigo tablets	ZC01^*^	270 mg (Tab)	Acute promyelocytic leukemia(APL)	July 2009
17	Astragalus polysaccharide for injection	ZC02^*^	250 mg (Inj)	Antineoplastic adjuvant drugs	August 2004
18	Shenyi capsule	ZC02^*^	10 mg (Cap)	Antineoplastic adjuvant drugs	April 2003

^▴^*ATC Code from the WHO Collaborating Center for Drug Statistics Methodology*.

^*^Medicine classification and codes for basic medical insurance.

^**#**^*NMPA, National Medical Products Administration of China*.

In data collection, first, we identified public hospitals with continuous monthly records of the 18 anticancer drugs from January 2015 to December 2019, which left us with 887 hospitals located in 10 eastern provinces, 6 central provinces, 12 western provinces and 3 north-eastern provinces. Of the 887 total hospitals, 241 (21.17%) were secondary, while 646 (72.83%) were tertiary. Then, we collected monthly average of drug availability and cost from January 2015 to December 2019 for the bundle of 18 drugs, for each drug, and for Western Medicines and Traditional Chinese Medicines separately. At last, we were able to collect yearly average of availability and cost of each drug from 2015 to 2019 for tertiary and secondary hospitals separately, and for different regions separately.

### Measurements

In line with the WHO/HAI methodology and previous research (31, 32), we measured the accessibility of all 18 anticancer drugs in terms of availability and cost.

#### Availability

For each drug *i*, we defined availability as the proportion of hospitals in which it was available. This was calculated as the percentage of hospitals where drug could be found in our sample, as follows (33).


$$\begin{array}{l} Availability\;\left(i\right)\\ =\frac{number\;of\;hospitals\;where\;drug\left(i\right)\;can\;be\;found}{total\;number\;of\;hospitals\;in\;the\;sample}\times 100\% \end{array}$$

#### Cost

We used the defined daily dose cost (DDDc) to reflect the cost and financial burden of each drug *i*. DDDc is the average daily cost of the drug used by the patients, which is calculated based on the defined daily dose system (DDDs). DDDs is the drug consumption volume divided by the defined daily dose (DDD), as recommended by the WHO, clinical application guidelines, and drug package inserts approved by the National Medical Products Administration of China (NMPA). It is calculated as follows (34).


$$\begin{array}{lll} DDDs\left(i\right) & = & \frac{total\;consumption\;volume\;of\;anticancer\;drug\left(i\right)}{DDD\left(i\right)}\\ DDDc\left(i\right) & = & \frac{total\;consumption\;sum\;of\;anticancer\;drug\left(i\right)}{DDDs\left(i\right)} \end{array}$$

### Statistical Analysis

#### Interrupted Time Series

The ITS employs a quasi-experimental design to evaluate the longitudinal effects of time-delimited interventions. In statistical terms, a segmented regression analysis of interrupted time can be used to assess the extent to which an intervention has changed an outcome of interest, either transiently or in the long-term context (35). We used the ITS to analyze the impact of NRDLN on the availability and cost of 18 anticancer drugs overall and separately, as well as Western Medicines and Traditional Chinese Medicines separately. We regarded July 2017 as the fixed intervention time point. The ITS regression model with one intervention is typically formulated as follows:


$$\begin{array}{l} Y_{t}=\beta_{0}+\beta_{1}*time_{t}+\beta_{2}*NRDLN_{t}+\beta_{3}*time\;after\;NRDLN_{t}+\varepsilon_{t} \end{array}$$

Where *Y*_*t*_ is the independent outcome variable (monthly average availability and DDDc in month *t*), *time*_*t*_ refers to a continuous variable indicating the number of months at time *t* from the beginning of the observation period (January 2015–June 2017), *NRDLN*_*t*_ is a dummy variable for time *t* occurring either before (*NRDLN*_*t*_ = 0) or after (*NRDLN*_*t*_ = 1) NRDLN implementation in July 2017, *time after NRDLN*_*t*_ is a continuous variable indicating the number of months at time *t* after NRDLN implementation (July 2017–December 2019), which was set at 0 before July 2017. β_0_estimates the level of the outcome at the beginning of the observation period, β_1_ estimates the linear trend during the pre-intervention period, and β_3_ estimates the change in trend in the outcome after NRDLN implementation compared with baseline. ε_*t*_ is error term at time *t* (36).

Two robustness checks were conducted including the Augmented Dickey-Fuller test and controlled ITS. The Augmented Dickey-Fuller test was to test the stationary of time series (35, 37). Controlled ITS was to control for potential seasonal fluctuations in series (38). Monthly dummy variables were usually included to avoid spurious associations (39, 40).

#### Multilevel Growth Curve Model

With yearly data stratified by region or hospital level, we employed a standard two-level growth curve model to estimate changes in the average level and growth rate of availability and cost. Despite the same linear growth assumption, different parameters of growth curves are allowed to different drugs in multilevel modeling (41). The specification is as follows:


$$\begin{array}{l} Y_{it}=\lambda_{0i}+\lambda_{1i}\left(year_{it}-2017\right)+\lambda_{2t\;}\\ NRDLN_{it}+\lambda_{3t}\;year\;after\;NRDLN_{it}+e_{it} \end{array}$$

Where *Y*_*it*_ is the yearly average availability and DDDc of drug *i* in year *t, year*_*it*_ is centered to 2017, and both *NRDLN*_*it*_ and *year after NRDLN*_*it*_ for drug *i* are the same as above. We fit different intercepts and slopes for each drug, thus allowing for variation between and within drugs. The intercept and slope equations are as follows:


$$\begin{array}{lll} \lambda_{0i} & = & v_{00}+u_{0i}\\ \lambda_{1i} & = & v_{10}+v_{11}NRDLN_{it}+v_{12}\;year\;after\;NRDLN_{it}+u_{1i} \end{array}$$

#### Statistical Analysis Software

We conducted the ITS analysis in SAS V 9.4 and multilevel growth curve models in StataSE V 17.0, with statistical significance determined at *P* < 0.05.

## Results

### Descriptive Statistics

#### Monthly Availability and Cost

**Figure 1:**
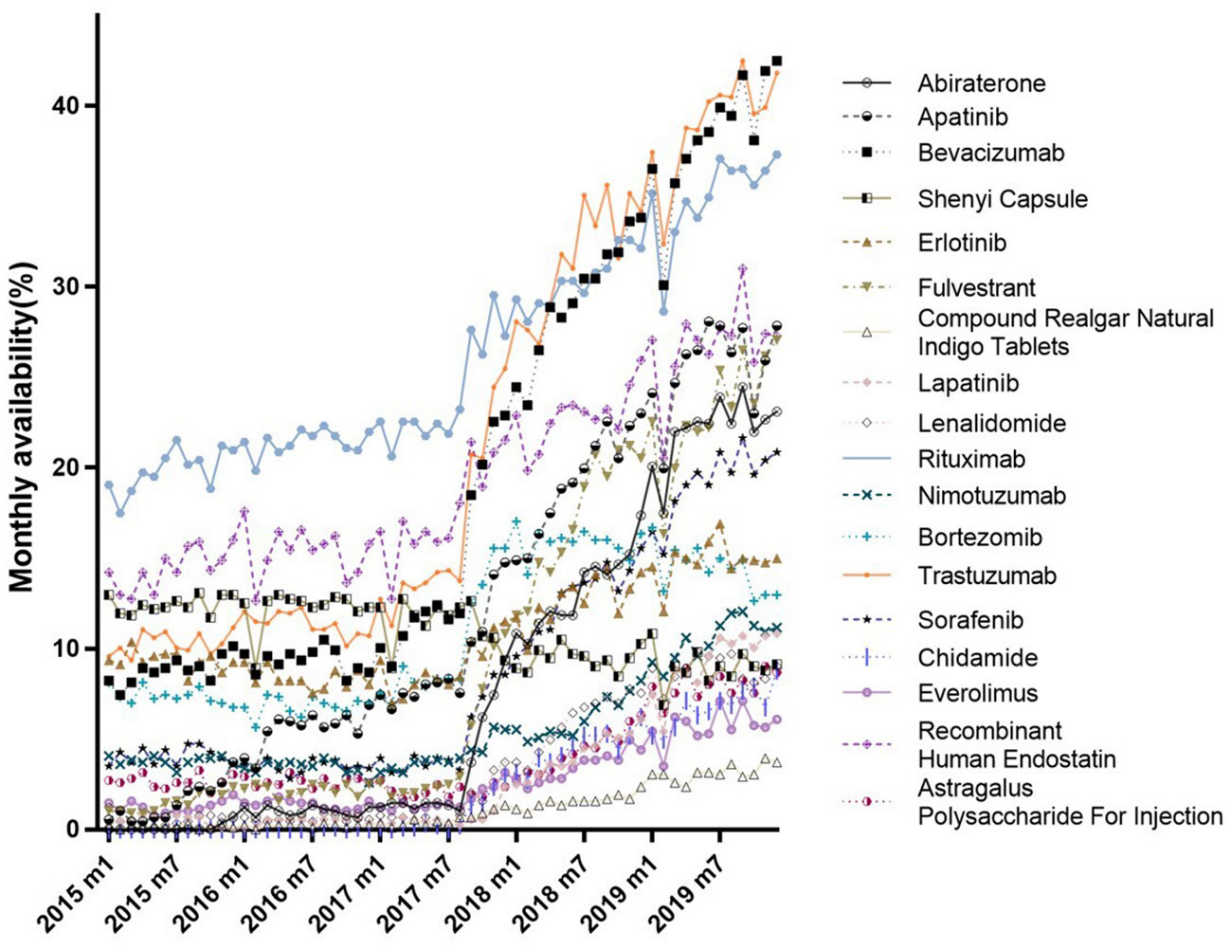
Trends in monthly availability of all 18 investigated drugs (January 2015–December 2019).

Figure 1 shows trends in the monthly availability of each 18 anticancer drug from January 2015 to December 2019. There was a fluctuating uptrend, with the exception of the Shenyi Capsule. There were obvious changes in the availabilities of most drugs between July and September 2017; for example, see Abiraterone, Trastuzumab, and Bevacizumab. While the Compound Realgar Natural Indigo Tablets, Everolimus, Astragalus Polysaccharides for Injection, and Chidamide had lower availabilities, those for Bevacizumab, Trastuzumab, and Rituximab were as high as 42.50, 41.83, and 37.32%, respectively, but none were higher than 45% until December 2019 (Supplementary Table 1).

**Figure 2:**
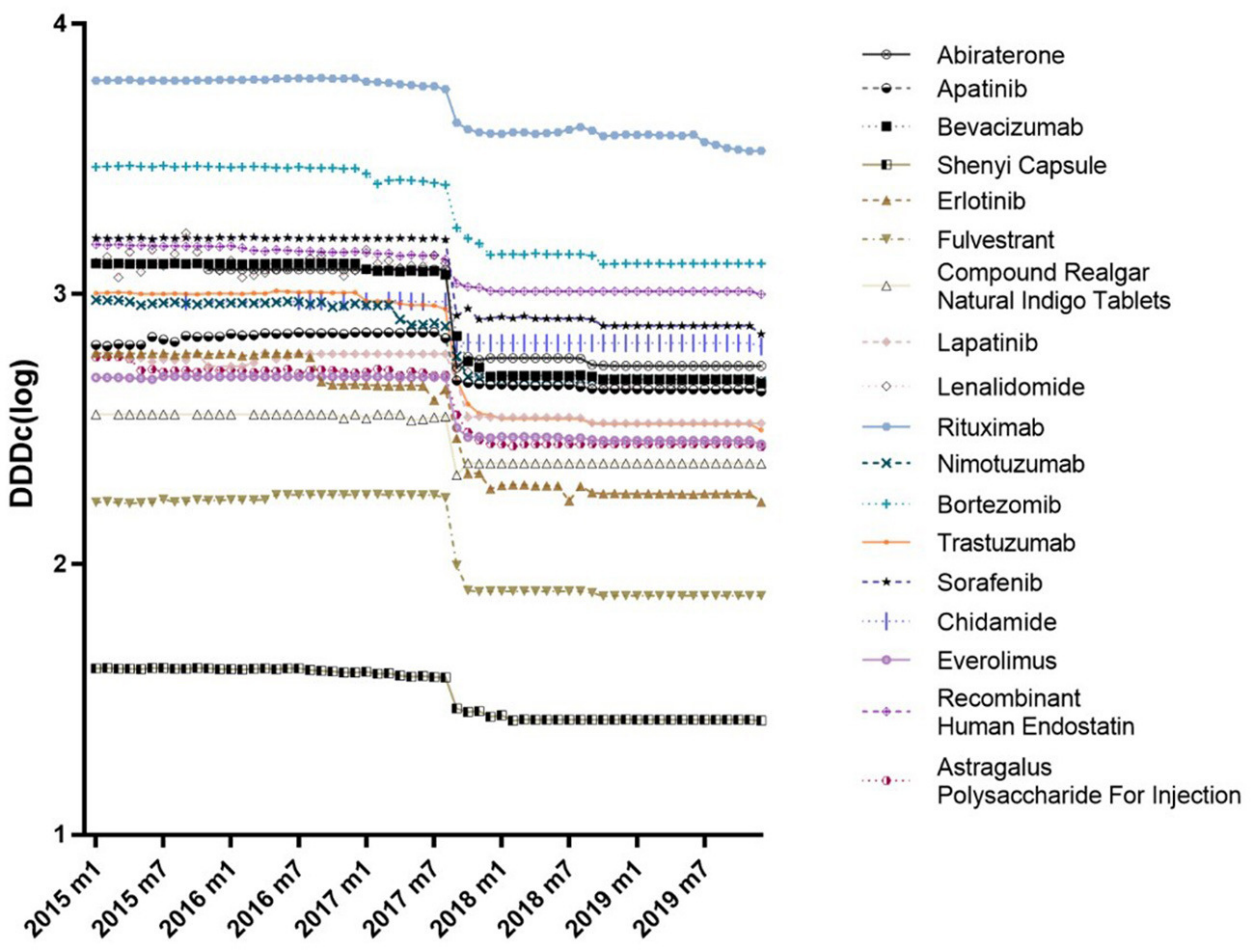
Trends in monthly DDDc of all 18 investigated drugs (January 2015–December 2019).

Figure 2 shows trends in the monthly DDDc for each 18 anticancer drugs from January 2015 to December 2019. The DDDc of Rituximab was the highest, while that for the Shenyi Capsule was the lowest. For each drug, there was a consistent downtrend in DDDc between July and September 2017. All the reductions in DDDc from January 2015 to December 2019 were above 32% (Supplementary Table 2). Eight drugs had a drop more than 50% in DDDc, while four drugs dropped more than 60% (i.e., Erlotinib, Trastuzumab, Bevacizumab, and Lenalidomide).

As suggested by Augmented Dickey-Fuller test results, our time series data of monthly availability and DDDc were stationary.

#### Yearly Availability and Cost

Based on the descriptive results in Table 2, overall availability was increasing from 2015 to 2019. In 2019, 30.47% of the surveyed hospitals could provide all 18 drugs, compared to 10.49% in 2015. The availabilities of surveyed drugs in Eastern, Central, Western, and Northeastern China had, respectively increased to 37.65, 26.96, 26.09, and 21.46% by the end of 2019. Compared with 14.59% in secondary hospitals, the availability of 18 drugs was 36.39% in tertiary hospitals. In 2019, the 15 Western Medicines (33.86%) were more widely available than the 3 Traditional Chinese Medicines (13.53%).

**Table 2:** Descriptive results of both overall yearly availability and DDDc in the nationwide, regional, hospital-level, and drug category contexts for each observed year (2015–2019).

		**Availability (%)**					**DDDc (CNY)**				
**Year**		**2015**	**2016**	**2017**	**2018**	**2019**	**2015**	**2016**	**2017**	**2018**	**2019**
Nationwide		10.49	10.89	16.34	24.70	30.47	527.93	545.94	504.20	421.86	401.87
Drug categories	15 Western medicines	10.87	11.50	17.91	27.36	33.86	1406.49	1379.14	942.92	501.43	447.86
	3 Traditional Chinese medicines	8.61	7.85	8.49	11.42	13.53	53.90	54.20	43.35	45.87	65.28
Regions	Eastern China	13.21	13.95	22.40	31.93	37.65	634.18	635.02	598.93	444.29	421.11
	Central China	9.57	9.51	14.06	22.06	26.96	533.90	473.13	350.87	351.31	344.45
	Western China	9.61	9.69	12.42	18.53	26.09	250.59	324.06	357.20	476.54	435.73
	Northeastern China	10.16	9.31	10.11	16.31	21.46	358.68	445.24	441.89	357.86	357.85
Hospital levels	Tertiary hospitals	13.24	13.75	20.20	29.91	36.39	527.65	546.65	504.83	422.74	402.41
	Secondary hospitals	4.03	3.87	6.35	10.74	14.59	542.28	505.84	466.22	384.83	382.59

For all 18 anticancer drugs, nationwide DDDc gradually decreased from 527.93 CNY in 2015 to 401.87 CNY in 2019, with a reduction of 23.88%. The DDDc decreased in Eastern and Central China, whilst increased in Western China. Meanwhile, DDDc was essentially flat in Northeastern China. As for tertiary and secondary hospitals, DDDc was 402.41 CNY and 382.59 CNY in 2019, respectively. Finally, the 15 Western Medicines had much higher DDDc (447.86 CNY) than the 3 Traditional Chinese Medicines (65.28 CNY).

### Main Results

#### Changes in Availability

**Figure 3:**
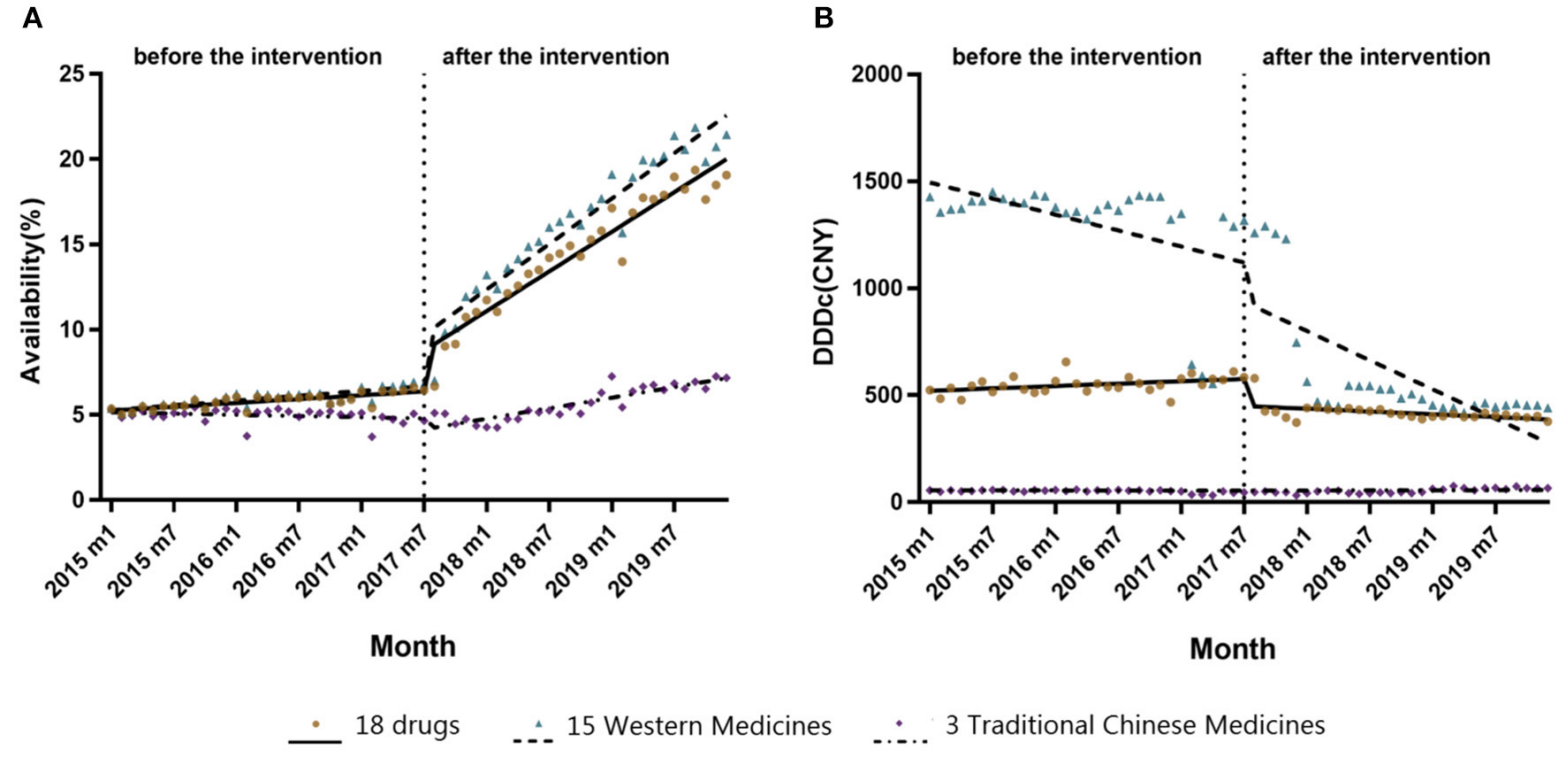
**(A)** Changes in monthly availability (%) of the 15 Western Medicines, 3 Traditional Chinese Medicines, and all 18 drugs combined both before and after NRDLN implementation in July 2017. **(B)** Changes in monthly DDDc (CNY) of the 15 Western Medicines, 3 Traditional Chinese Medicines, and all 18 drugs combined both before and after NRDLN implementation in July 2017.

Table 3 shows the results of the ITS analysis for monthly availability and DDDc. There was a slight increase in the availability of all 18 anticancer drugs prior to the intervention (β_1_ = 0.0368, *P* < 0.05). After NRDLN, there was a significant increase in both the intercept (β_2_ = 2.1126, *P* < 0.001) and slope (β_3_ = 0.3656, *P* < 0.001). In other words, the increasing availability trend accelerated after NRDLN implementation, reaching nearly 10 times higher than baseline. As shown in Figure 3A, there was also an interruption point in July 2017; that is, there was an instant increase in the availability of all 18 anticancer drugs, followed by a continually increasing trend thereafter. It suggested that the NRDLN had a substantial impact on the availability of anticancer drugs.

**Table 3:** ITS results of the impacts of NRDLN on monthly availability and DDDc for all 18 drugs, with a breakdown for the 15 Western Medicines and 3 Traditional Chinese Medicines (2015–2019).

	**Availability**				**DDDc**			
	**Estimate**	**SE**	***t***	***P*-Value**	**Estimate**	**SE**	***t***	***P*-Value**
**18 drugs**								
Intercept β_0_	5.2262	0.2785	18.76	<0.0001***	519.6131	14.2226	36.53	<0.0001***
Baseline trend β_1_	0.0368	0.0157	2.35	0.0226*	1.8133	0.8011	2.26	0.0275*
Level change β_2_	2.1126	0.3844	5.50	<0.0001***	−108.7213	19.6293	−5.54	<0.0001***
Trend change β_3_	0.3656	0.0222	16.48	<0.0001***	−4.8332	1.1330	−4.27	<0.0001***
**15 western medicines**								
Intercept β_0_	5.2411	0.3310	15.84	<0.0001***	1521	79.7515	19.07	<0.0001***
Baseline trend β_1_	0.0463	0.0186	2.49	0.0159*	−13.8117	4.4923	−3.07	0.0033**
Level change β_2_	2.6534	0.4568	5.81	<0.0001***	−108.5263	110.0690	−0.99	0.3284
Trend change β_3_	0.4165	0.0264	15.80	<0.0001***	−11.3325	6.3531	−1.78	0.0799
**3 traditional Chinese medicines**								
Intercept β_0_	5.1503	0.1595	32.29	<0.0001***	57.5658	2.5532	22.55	<0.0001***
Baseline trend β_1_	−0.0109	0.0090	−1.21	0.2314	−0.3839	0.1438	−2.67	0.0099**
Level change β_2_	−0.5924	0.2201	−2.69	0.0094**	−5.6677	3.5238	−1.61	0.1134
Trend change β_3_	0.1108	0.0127	8.72	<0.0001***	1.3124	0.2034	6.45	<0.0001***

*P < 0.05, **P < 0.01, ***P < 0.001.

#### Changes in Cost

In Table 3 and Figure 3B, we observed a statistically significant interruption point in the time series of DDDc in July 2017. There was a slow rise in DDDc for all 18 anticancer drugs before July 2017 (β_1_ = 1.8133, *P* < 0.05). At the time of policy implementation, there was a significant and substantial decrease in the DDDc (β_2_ = −108.7213, *P* < 0.001). After that, there was a significant decline in the slope (β_3_ = −4.8332, *P* < 0.001). The implementation of NRDLN catalyzed a downward trend in the cost of all 18 anticancer drugs.

Our results are robust using controlled ITS (Supplementary Table 3).

### Stratification Analyses Results

#### Different Drug Categories

As shown in Table 3, there were significant increases in the intercept (β_2_ = 2.6534, *P* < 0.001) and regression slope (β_3_ = 0.4165, *P* < 0.001) pertaining to the availability of the 15 Western Medicines after NRDLN implementation. As shown in Figure 3A, there was a clear and immediate rise at the time of July 2017, followed by a steady and significant monthly increase. Meanwhile, there was a significant decrease in the intercept (β_2_ = −0.5924, *P* < 0.01), as well as a significant increase in the slope (β_3_ = 0.1108, *P* < 0.001) pertaining to the availability of the 3 Traditional Chinese Medicines. In addition, no significant instant decrease in DDDc was observed. A significant increase in slope was only reported in the DDDc of Traditional Chinese Medicines (β_3_ = 1.3124, *P* < 0.001).

#### Different Regions

As shown in Table 4, prior to 2017, there was a significant rise in yearly availability in Eastern China (λ_1_ = 0.228, *P* < 0.05). After 2017, there were significant increases in the intercepts of yearly availability for Eastern, Central, and Western China, as well as in the slopes for Central, Western, and Northeastern China. Western China experienced the highest increases in both yearly availability (λ_2_ = 0.723, *P* < 0.01) and upward trend (λ_3_ = 0.571, *P* < 0.001). Meanwhile, there was a significant decrease in yearly DDDc in terms of intercepts and slopes for all regions. The greatest decrease in yearly DDDc was found in Central China (λ_2_ = −0.436, *P* < 0.001), while the sharpest downward trend was found in Northeastern China (λ_3_ = −0.220, *P* < 0.001).

**Table 4:** Growth curve model estimates of yearly availability and DDDc stratified by region and hospital level (2015–2019).

	**Eastern China**	**Central China**	**Western China**	**Northeastern China**	**Tertiary hospital**	**Secondary hospital**
**Model A: log (availability)**						
*n*	90	87	82	86	90	82
*λ_1_*	0.228*	0.125	−0.008	0.106	0.195	0.138
*λ_2_*	0.523***	0.699***	0.723**	0.026	0.501***	0.591*
*λ_3_*	0.095	0.352**	0.571***	0.416**	0.191*	0.456*
Constant	2.352***	1.568***	1.299***	1.900***	2.244***	0.612
**Model B: log (DDDc)**						
*n*	90	87	82	86	90	81
*λ_1_*	−0.008	−0.007	−0.023	−0.007	−0.010	0.005
*λ_2_*	−0.347***	−0.436***	−0.347***	−0.339***	−0.347***	−0.406***
*λ_3_*	−0.182***	−0.137***	−0.188**	−0.220***	−0.184***	−0.186***
Constant	6.644***	6.658***	6.687***	6.697***	6.650***	6.706***

*P < 0.05, **P < 0.01, ***P < 0.001.

#### Different Hospital Levels

In Table 4, we observed an insignificant time trend in both yearly availability and DDDc in the tertiary and secondary hospitals prior to 2017. However, there was a significant increase in yearly availability and significant decrease in yearly DDDc in all hospital after 2017. The tertiary hospitals had a smaller increase in the slope of yearly availability (λ_3_ = 0.191, *P* < 0.05) compared to that of secondary hospitals (λ_3_ = 0.456, *P* < 0.05). In addition, secondary hospitals had a slightly larger instant reduction in yearly DDDc (λ_2_ = −0.406, *P* < 0.001) than that of tertiary hospitals (λ_2_ = −0.347, *P* < 0.001).

## Discussion

This study investigated the impact of NRDLN policy on the availability and cost of 18 anticancer drugs based on procurement data from a nationally representative sample of hospitals. As expected, the NRDLN increased the availability and decreased the cost of anticancer drugs. This finding was similar to some recent studies on specific regions of China (25–27, 42, 43). It is indicated that the implementation of NRDLN exerts a strong positive effect on the accessibility of anticancer drugs in China. This effect has not only short-term but also long-term significances.

### Impact of NRDLN Policy on Availability

As for availability, our results showed an instant increase after NRDLN implementation, followed by an even sharper rise. Regardless of the drug category, hospital level, and region, a greater number of hospitals were able to provide the investigated anticancer drugs after NRDLN policy, thus entailing greater access for patients.

Despite the increased availability of most anticancer drugs, the overall availability of most anticancer drugs in China remained low (<30%). The highest availability level was less than 50% by the end of 2019, which is unsatisfactory (44). This reinforces reports from similar studies (22, 29). There are many possible reasons for this. For example, anticancer drug costs are higher than other drugs; meanwhile, there are problems with the total pre-payment of medical insurance and insufficient procurement incentives for hospitals, which can weaken policy effects (45, 46). This situation can be improved through the implementation of additional supportive measures and coordination with other policies.

We observed some variations in drug availability. First, drugs for cancers with higher incidence rates in China (NSCLC, colorectal cancer, breast cancer, gastric cancer, and NHL) were generally of higher availability. This is consistent with some previous research wherein cancer prevalence affects the availability of anticancer drugs (28). Second, drug availability was also impacted by whether and when these drugs received NMPA market approval. For example, if there was a long market approval time before market launch, drug availability tended to be lower, which was the case for Chidamide. Lastly, anticancer drugs with a larger cost decrease were more available. For instance, in our study, Bevacizumab and Trastuzumab had a decrease of more than 60% in cost, along with an increase of 416.40 and 336.50% in availability, respectively by 2019.

In addition, it was found that the availability of 15 Western Medicines increased at a higher rate than 3 Traditional Chinese Medicines. The Traditional Chinese Medicines experienced a short-term drop in availability, but a long-term increase after NRDLN implementation. Although Traditional Chinese anticancer medicines are mainly intended for anticancer and adjuvant indication applications, they are added to the national reimbursement drug list as a powerful means of cancer therapy. However, most studies have ignored the utilization data on traditional Chinese anticancer medicines, with some remaining unprovided at a number of hospitals (28).

Furthermore, we found that availability was the highest in Eastern China, followed by Central and Western China, and lowest in Northeastern China after NRDLN implementation. These regional differences in availability are consistent with previous research on essential drugs (47), which probably leads to inequity in drug accessibility. We also found varied increases in availability from 2015 to 2019 in different regions. The highest annual increase was found in Western China, which might be due to low initial availability, or different regional policy responses. Moreover, there were similar results for hospital level. Compared to tertiary hospitals, availability was lower in secondary hospitals, whereas the slope increase was higher in secondary hospitals. Overall, these findings suggest that regional and hospital-level disparities in the availability of anticancer drugs have gradually decreased by NRDLN implementation.

### Impact of NRDLN Policy on Cost

As for cost, we found an instant decrease after NRDLN implementation, followed by a continuous downward trend, both of which were significant. There was a significant decline of 108.7213 CNY in DDDc in July 2017, which was equivalent to an average monthly cost reduction of 3261.60 CNY for patients. The increasing trend of drug cost was flipped over by the implementation of NRDLN. After July 2017, DDDc fell at a faster rate, equal to a monthly 145.00 CNY downward trend in anticancer drug costs, thus decreasing expenditures for patients. This is in line with some recent studies reporting that the NRDLN has helped to lower the prices of anticancer drugs, and is the direct reason for the significant reduction in average daily costs. DDDc also represented a comparable price of anticancer drugs (25, 27, 43, 48).

It should be noted that DDDc of anticancer drugs was decreasing from 2017 to 2019, same as the national trend of all drugs in China (44). The DDDc of 18 anticancer drugs in our study was over twice higher than that of all the anticancer drugs, or around 30 times higher than that of all drugs in 2019. This might be due to changes in both the variety and structure of anticancer drugs as well as the relatively high prices and expenses of innovative anticancer drugs covered by the NRDLN.

Our results showed significantly decreased DDDc associated with NRDLN in all regions, as well as in the tertiary and secondary hospitals. Contrary to the results on availability, changes in DDDc did not have significant regional or hospital-level disparities, which could be seen as a piece of evidence that the negotiated price of anticancer drugs has been well and equally adopted after NRDLN.

### Suggestions

Based on our findings, the NRDLN is an effective way to promote patients' access to anticancer drugs. However, there is still a gap between current conditions and ultimate expectations for improving the accessibility to anticancer drugs. More efforts are needed to further enhance the effects of the NRDLN.

First, there should be shorter market approval duration for internationally listed innovative anticancer drugs in China (1). This requires a quicker market approval process for generic and biosimilar anticancer drugs with high effectiveness through the NRDLN. Second, unequal accessibility in different drug categories, regions, and hospitals should be addressed by implementing preferential policies, ensuring rational budget allocations, monitoring and standardizing the anticancer drug prescription structure, and objective policy evaluations in China. Lastly, there should be stronger supportive measures and enhanced coordination with other policies, such as sufficient incentives for hospitals to purchase and prescribe NRDLN drugs, coordination among multilevel medical security systems, value-based pricing and payments, and a dual channel for NRDLN drugs (49).

### Limitations

This study also had some limitations. First, the CMEI database provides information on the clinical use of anticancer drugs in Chinese hospitals. Although closely related, this is not a direct substitute for information on anticancer drug prescriptions at hospitals or drug usage among individual patients. Second, we did not consider data from private hospitals or pharmacies. Third, we quantified changes in 18 anticancer drugs affected by the NRDLN, which did not represent other anticancer drugs or other NRDLN drugs. Finally, we used availability and daily costs to evaluate accessibility. Further research is needed in other aspects such as whether patients can afford anticancer drugs.

## Conclusion

The NRDLN constitutes a national determination and effort to enhance population health in China. This study provides evidence that the implementation of NRDLN improves the availability and reduces the cost of some anticancer drugs in China. It contributes to promoting accessibility of anticancer drugs, as well as relieving regional or hospital-level disparities. However, there are still challenges to benefit more patients sufficiently and equally. It requires more policy efforts and collaborative policy combination.

## Data Availability Statement

The original contributions presented in the study are included in the article/Supplementary Material, further inquiries can be directed to the corresponding author/s.

## Author Contributions

HZ, JZ, and QW conceptualized and undertook the analyses, and wrote the original draft of the manuscript. HZ, LS, and QW designed the study. YZ provided the data. YZ, JZ, LS, and CL contributed to the analysis method. QW and YH critically reviewed and modified the initial and subsequent drafts. All authors have read and approved the final manuscript for publication.

## Funding

This study was funded by National Social Science Foundation of China (No.19AZD013), Natural Science Foundation of Heilongjiang Province, China (No.YQ2019G003), and Humanities and Social Science College Program of Harbin Medical University (No.HMURW20210101). This study was also funded by a co-operative project between Health Management College, Harbin Medical University and Science and Technology Development Center of the Chinese Pharmaceutical Association.

## Conflict of Interest

The authors declare that the research was conducted in the absence of any commercial or financial relationships that could be construed as a potential conflict of interest.

## Publisher's Note

All claims expressed in this article are solely those of the authors and do not necessarily represent those of their affiliated organizations, or those of the publisher, the editors and the reviewers. Any product that may be evaluated in this article, or claim that may be made by its manufacturer, is not guaranteed or endorsed by the publisher.
