# Supplementary material for: Impact of the National Reimbursement Drug List Negotiation Policy on Accessibility of Anticancer Drugs in China: An Interrupted Time Series Study
Source: Front Public Health. 2022 Jul 1;10:921093. doi: 10.3389/fpubh.2022.921093 (PMC9283976; doi:10.3389/fpubh.2022.921093)
Supplement: Supplementary file 3 [file Table_3.DOCX]

**Supplementary Table 3.** Controlled ITS analyses results of impact on monthly availability and DDDc of 18 anticancer drugs

|  | **Availability** | | | | **DDDc** | | | |
| --- | --- | --- | --- | --- | --- | --- | --- | --- |
|  | **Estimate** | **SE** | **t** | **p** | **Estimate** | **SE** | **t** | **p** |
| **18 drugs** | | | | | | | | |
| Intercept β0 | 5.4753 | 0.4067 | 13.46 | <.0001*** | 530.2185 | 20.3987 | 25.99 | <.0001*** |
| Baseline trend β1 | 0.0357 | 0.0148 | 2.41 | 0.0199* | 2.0444 | 0.7422 | 2.75 | 0.0085** |
| Level change β2 | 1.8310 | 0.3660 | 5.00 | <.0001*** | -110.858 | 18.3604 | -6.04 | <.0001*** |
| Trend changeβ3 | 0.3659 | 0.0207 | 17.66 | <.0001*** | -4.7228 | 1.0396 | -4.54 | <.0001*** |
| mon1 | -0.3137 | 0.4438 | -0.71 | 0.4833 | 3.1809 | 22.2581 | 0.14 | 0.8870 |
| mon2 | -0.5923 | 0.4426 | -1.34 | 0.1875 | 20.1463 | 22.2016 | 0.91 | 0.3690 |
| mon3 | 0.004976 | 0.4418 | 0.01 | 0.9911 | -11.0264 | 22.1580 | -0.50 | 0.6212 |
| mon4 | -0.8863 | 0.4412 | -2.01 | 0.0506 | -31.9231 | 22.1275 | -1.44 | 0.1560 |
| mon5 | -0.3662 | 0.4408 | -0.83 | 0.4105 | -34.9578 | 22.1102 | -1.58 | 0.1209 |
| mon6 | -0.2609 | 0.4407 | -0.59 | 0.5569 | -60.6885 | 22.1061 | -2.75 | 0.0087** |
| mon7 | 0.3328 | 0.4424 | 0.75 | 0.4557 | -10.9718 | 22.1884 | -0.49 | 0.6234 |
| mon8 | -1.0608 | 0.4412 | -2.40 | 0.0204* | 4.9210 | 22.1292 | 0.22 | 0.8250 |
| mon9 | -0.0513 | 0.4403 | -0.12 | 0.9077 | -15.5023 | 22.0831 | -0.70 | 0.4863 |
| mon10 | 0.0860 | 0.4396 | 0.20 | 0.8458 | -32.7975 | 22.0501 | -1.49 | 0.1439 |
| mon11 | -0.0199 | 0.4392 | -0.05 | 0.9640 | -10.6788 | 22.0302 | -0.48 | 0.6302 |
| **15 Western Medicines** | | | | | | | | |
| Intercept β0 | 5.5322 | 0.4985 | 11.1 | <.0001*** | 1583.0000 | 109.0692 | 14.51 | <.0001*** |
| Baseline trend β1 | 0.0452 | 0.0181 | 2.49 | 0.0164* | -14.5316 | 3.9685 | -3.66 | 0.0007** |
| Level change β2 | 2.3396 | 0.4487 | 5.21 | <.0001*** | -120.6548 | 98.1706 | -1.23 | 0.2254 |
| Trend changeβ3 | 0.4169 | 0.0254 | 16.41 | <.0001*** | -11.0975 | 5.5586 | -2.00 | 0.0520 |
| mon1 | -0.3935 | 0.5439 | -0.72 | 0.4732 | 51.1251 | 119.0116 | 0.43 | 0.6696 |
| mon2 | -0.7248 | 0.5425 | -1.34 | 0.1883 | 62.7913 | 118.7091 | 0.53 | 0.5994 |
| mon3 | -0.0416 | 0.5415 | -0.08 | 0.9392 | 92.8034 | 118.4761 | 0.78 | 0.4375 |
| mon4 | -1.0151 | 0.5407 | -1.88 | 0.0670 | 94.9136 | 118.3132 | 0.80 | 0.4266 |
| mon5 | -0.4640 | 0.5403 | -0.86 | 0.3950 | 123.1877 | 118.2206 | 1.04 | 0.3030 |
| mon6 | -0.3356 | 0.5402 | -0.62 | 0.5376 | 17.1739 | 118.1984 | 0.15 | 0.8851 |
| mon7 | 0.3504 | 0.5422 | 0.65 | 0.5214 | -78.9852 | 118.6389 | -0.67 | 0.5090 |
| mon8 | -1.1093 | 0.5408 | -2.05 | 0.0461* | -241.3546 | 118.3224 | -2.04 | 0.0473* |
| mon9 | -0.0571 | 0.5396 | -0.11 | 0.9162 | -231.3159 | 118.0756 | -1.96 | 0.0563 |
| mon10 | 0.0963 | 0.5388 | 0.18 | 0.8589 | -232.9933 | 117.899 | -1.98 | 0.0543 |
| mon11 | -0.033 | 0.5383 | -0.06 | 0.9513 | -15.8766 | 117.7929 | -0.13 | 0.8934 |
| **3 Traditional Chinese Medicines** | | | | | | | | |
| Intercept β0 | 5.1883 | 0.2092 | 24.8 | <.0001  *** | 56.4281 | 4.2241 | 13.36 | <.0001*** |
| Baseline trend β1 | -0.0117 | 0.0076 | -1.54 | 0.1304 | -0.3585 | 0.1537 | -2.33 | 0.0242* |
| Level change β2 | -0.7131 | 0.1883 | -3.79 | 0.0004** | -7.9103 | 3.8021 | -2.08 | 0.0432* |
| Trend changeβ3 | 0.1111 | 0.0107 | 10.42 | <.0001*** | 1.3167 | 0.2153 | 6.12 | <.0001*** |
| mon1 | 0.0877 | 0.2283 | 0.38 | 0.7028 | 3.0525 | 4.6092 | 0.66 | 0.5112 |
| mon2 | 0.0703 | 0.2277 | 0.31 | 0.7589 | 2.0290 | 4.5975 | 0.44 | 0.6611 |
| mon3 | 0.2406 | 0.2273 | 1.06 | 0.2955 | 3.1714 | 4.5885 | 0.69 | 0.4930 |
| mon4 | -0.2426 | 0.2269 | -1.07 | 0.2908 | -0.3401 | 4.5822 | -0.07 | 0.9412 |
| mon5 | 0.1233 | 0.2268 | 0.54 | 0.5895 | 0.7163 | 4.5786 | 0.16 | 0.8764 |
| mon6 | 0.1135 | 0.2267 | 0.50 | 0.6191 | -2.3352 | 4.5777 | -0.51 | 0.6125 |
| mon7 | 0.2462 | 0.2276 | 1.08 | 0.2850 | 2.6650 | 4.5948 | 0.58 | 0.5648 |
| mon8 | -0.8161 | 0.2270 | -3.60 | 0.0008** | -2.0132 | 4.5825 | -0.44 | 0.6625 |
| mon9 | -0.0222 | 0.2265 | -0.10 | 0.9223 | 4.3266 | 4.5730 | 0.95 | 0.3491 |
| mon10 | 0.0353 | 0.2261 | 0.16 | 0.8768 | -0.8416 | 4.5661 | -0.18 | 0.8546 |
| mon11 | 0.0475 | 0.2259 | 0.21 | 0.8344 | -0.8938 | 4.562 | -0.20 | 0.8456 |

*p < 0.05, **p < 0.01, ***p < 0.001.
